# Thromboinflammation in dogs with virulent babesiosis

**DOI:** 10.3389/fvets.2026.1712262

**Published:** 2026-03-12

**Authors:** Amelia Goddard, Johan P. Schoeman, Andrew L. Leisewitz, Peter N. Thompson, Annemarie T. Kristensen

**Affiliations:** 1 Department of Companion Animal Clinical Studies, Faculty of Veterinary Science, University of Pretoria, Onderstepoort, South Africa; 2 Department of Clinical Sciences, Auburn University College of Veterinary Medicine, Auburn, AL, United States; 3 Department of Production Animal Studies, Faculty of Veterinary Science, University of Pretoria, Onderstepoort, South Africa; 4 Department of Veterinary Clinical Sciences, Faculty of Health and Medical Sciences, University of Copenhagen, Copenhagen, Denmark

**Keywords:** canine babesiosis, consumptive coagulopathy, hypofibrinolysis, immunothrombosis, platelet activation

## Abstract

**Introduction:**

*Babesia rossi* infection in dogs is associated with severe hemostatic dysregulation and systemic inflammation. The close interplay between innate immunity and coagulation in pathological states, including acute infections, may lead to thrombus formation, termed thromboinflammation, and has been proposed to underlie disease severity and poor outcomes. The objectives of this study were to investigate the presence of thromboinflammation in dogs with *B. rossi* infection by characterizing the overall hemostatic state, using thromboelastography (TEG) and plasma-based assays, and the systemic inflammatory response using acute phase reactants as markers, and further to determine whether, if present, thromboinflammation was associated with disease severity.

**Methods:**

Ninety-seven dogs naturally infected with *B. rossi* and 15 healthy control dogs were included. Diagnosis of babesiosis was confirmed by reverse line blot polymerase chain reaction. Blood samples were collected at presentation, prior to any treatment. Hemostatic function was assessed using TEG, routine coagulation assays, coagulation factor activity, and fibrinolysis markers. C-reactive protein (CRP) and serum amyloid A (SAA) were measured as markers of systemic inflammation. Twelve of the Babesia-infected dogs died (12%).

**Results:**

*Babesia*-infected dogs demonstrated hemostatic abnormalities marked by severe thrombocytopenia and a procoagulant state, including platelet activation, preserved clot strength, and elevated fibrinogen concentrations. Thromboelastography further revealed delayed clot initiation and propagation with maintained clot firmness, whereas conventional assays showed prolonged prothrombin time and activated partial thromboplastin time, along with markedly reduced coagulation factor activities in non-survivors. Minimal fibrinolysis was observed on TEG and was associated with elevated α2-antiplasmin activity and the presence of fibrin microthrombi in multiple organs. Markedly elevated CRP and SAA concentrations confirmed the simultaneous presence of systemic inflammation.

**Discussion:**

*B. rossi* infection in dogs induced thromboinflammation characterized by thrombocytopenia, platelet activation, coagulation factor consumption, fibrinolytic shutdown and systemic inflammation. Thromboinflammation may drive endothelial injury, microthrombosis, and an increased risk of organ failure and death. Future therapeutic interventions targeting thromboinflammation may improve patient outcomes.

## 1 Introduction

Immunothrombosis is an immune defense mechanism in which the host interplay between the innate immune system and hemostasis limits pathogen spread through the bloodstream by activating the coagulation system. This process involves coordination between innate immune cells expressing tissue factor (TF), mainly monocytes, neutrophils and platelets (1). Thromboinflammation, the aberrant and excessive activation of immunothombosis, is characterized by a systemic prothrombotic phenotype concurrent with systemic inflammation, contributing to the thrombotic complications in both acute and chronic infectious and non-infectious disease states (1–3).

During infection, immune cells detect pathogen-associated molecular patterns or damage-associated molecular patterns via surface pattern-recognition receptors. In response, monocytes and their microvesicles express TF at sites of infection, initiating the extrinsic coagulation pathway (1). All evidence to date indicates that TF and TF-bearing surfaces are the primary initiators of hemostasis *in vivo.* Tissue factor is an integral membrane protein normally separated from the blood by the vascular endothelium. The TF pathway is activated when the endothelium is disrupted and blood is exposed to extravascular cells, or when endothelial cells, neutrophils or monocytes are triggered to express TF on their cell membranes by pathogens (4–7). When activated, i.e., by activated platelets, neutrophils contribute by releasing neutrophil extracellular traps (NETs), which are web-like structures made of DNA, histones and neutrophil-derived granule proteins (i.e., myeloperoxidase and neutrophil elastase). These NETs enhance coagulation through direct activation of factor XII, binding to von Willebrand factor (vWF) to recruit platelets, activation of platelets via surface histones, inactivation of anticoagulants (e.g., tissue factor pathway inhibitor and thrombomodulin), and binding to TF to enhance the extrinsic coagulation pathway (1–3, 8).

Vascular health is maintained through the anti-inflammatory and antithrombotic properties of endothelial cells. During systemic inflammation, vascular endothelial cells become activated or damaged, leading to the loss of their antithrombotic properties. This shift is characterized by decreased production of nitric oxide and prostacyclin, increased TF expression, thrombomodulin shedding, and enhanced release of vWF. These activated endothelial cells engage with both leukocytes and platelets, significantly contributing to the thromboinflammatory response. Fibrinolysis is also suppressed during thromboinflammation, resulting in impaired clot resolution (2, 3, 9).

A variety of infectious and inflammatory conditions in humans and animals are associated with hemostatic activation, including cardiovascular disease, trauma, SARS-CoV-2 infection (COVID-19), canine parvoviral enteritis, and babesiosis (2, 3, 10–13). Canine babesiosis is a severe intra-erythrocytic, tick-borne disease and a major cause of morbidity and mortality globally. The clinical presentation in dogs with *Babesia rossi* infection varies, with complications including hemoconcentration, disseminated intravascular coagulation (DIC), neurological signs, icterus, hepatopathy, immune-mediated hemolytic anemia, acute kidney injury, pancreatitis, and pulmonary edema (14–16). These severe forms are believed to result from an exaggerated inflammatory host response to the parasitemia, leading to systemic damage beyond that caused by hemolytic anemia and hypoxemia (17).

The severe systemic inflammatory host response, characteristic of *B. rossi* infection in dogs, contributes significantly to disease severity and outcome (18, 19). This response is accompanied by marked disturbances in hemostasis, including platelet activation, preserved clot strength despite thrombocytopenia, and consumptive coagulopathy with microthrombi formation in capillaries in severe cases, consistent with DIC (13, 15, 20–23). In both human and veterinary medicine, DIC is a major predictor of poor outcome (24, 25). Increasing evidence indicates that inflammatory and hemostatic pathways are tightly interlinked in this disease, suggesting the presence of a thromboinflammatory state rather than isolated abnormalities of either system.

Traditional plasma-based coagulation assays, such as prothrombin time (PT) and activated partial thromboplastin time (aPTT), can localize a hemostatic bleeding defect to either the intrinsic or extrinsic pathway of the coagulation cascade. However, the results do not necessarily correlate with the clinical phenotype, as these assays are performed on platelet-poor plasma, reflecting only the initiation phase of coagulation. As a result, they fail to account for the role of cellular elements in hemostasis. These assays are also limited by their inability to evaluate the rate of clot formation, overall clot strength, and the rate and degree of clot dissolution (26, 27). Alternatively, visco-elastic assays, including thromboelastography (TEG), provide a more holistic *in vitro* assessment of the dynamic process of clot formation and resolution. Except for the endothelium, visco-elastic assays include all components of hemostasis. These assays quantify the kinetics of clot development, the mechanical properties of the clot, and the subsequent degradation via fibrinolysis, providing detailed insights into coagulation and fibrinolytic activity across the entire clotting cascade. Importantly, this technology is also performed on whole blood, allowing for the incorporation of cellular elements essential to hemostasis (28–30). The velocity curve is a computed parametric derivative of a conventional TEG profile. The velocity curve enables quantification of clot propagation kinetics, including the maximum rate of thrombus generation (MRTG), the time to maximum rate of thrombus generation (TMRTG) and total thrombus generation (TG) (30).

Although systemic inflammation and coagulation dysfunction have been described separately in canine babesiosis, their integrated expression and relationship to disease severity remain incompletely understood. This study therefore aimed to investigate the presence of thromboinflammation in dogs infected with *B. rossi* by characterizing the overall hemostatic state using TEG and plasma-based coagulation assays, alongside assessment of the systemic inflammatory response using acute phase reactants. A further objective was to determine whether thromboinflammation, if present, was associated with disease severity. We hypothesized that dogs with *B. rossi* infection constitute a naturally occurring model of thromboinflammation, with more pronounced abnormalities in non-survivors.

## 2 Materials and methods

This prospective, observational study included client-owned dogs, naturally infected with *B. rossi* that presented for veterinary care to an academic hospital between October 2011 and April 2013. The research protocol was approved by the Research and Animal Ethics Committee of the University of Pretoria (V055-11). An initial diagnosis of infection with babesiosis was made through the recognition of commensurate clinical signs and demonstration of intra-erythrocytic trophozoites on stained thin blood smears, and was later confirmed as *B. rossi* mono-infection by reverse line blot polymerase chain reaction (RLB-PCR).

### 2.1 Study design

Dogs of either sex and of any breed were eligible for inclusion in the study provided they were ≥12 weeks of age, weighed ≥5 kg, and had a demonstrable parasitemia. Dogs were excluded if they were subsequently proven by RLB-PCR to be infected with *B. vogeli* or *Ehrlichia canis*, or euthanized for reasons other than poor prognosis. Dogs were also excluded if any concurrent inflammatory disease conditions (i.e., any obvious infections, wounds, or any signs of trauma), any known cardiac disease or cancer were present. Dogs receiving anti-inflammatory medication either at presentation or within 4 weeks prior to presentation were also excluded. All the dogs included in the study received standard care for canine babesiosis, which included antibabesial treatment with diminazene aceturate (Berenil RTU 0.07 g/mL, MSD Animal Health, Kempton Park, South Africa) at 3.5 mg/kg, and transfusion with packed red cells and intravenous fluids as needed. In addition, any complications were managed at the discretion of the attending clinician. Outcome was recorded as short-term survival (i.e., survival until discharge) or death/euthanasia due to poor prognosis. A complete post mortem examination was performed and tissue samples were collected in 10% formalin for routine hematoxylin and eosin processing in all non-surviving or euthanized cases. The control dogs consisted of 15 healthy, client-owned dogs admitted for routine ovariohysterectomy, castration, or blood donation. These dogs were deemed healthy based on history, a full physical examination, peripheral blood smear evaluation, complete blood count (CBC), and full biochemistry profile, as well as RLB-PCR to rule out infection.

### 2.2 Sample collection

At presentation and before any treatment, a serum sample (4 mL vacutainer tube; BD Biosciences, New Jersey, United States), sodium citrate sample (4 mL vacutainer tube) and EDTA sample (4 mL vacutainer tube) were collected from the jugular vein of each patient and control with a 21-gage needle by careful venipuncture with vacuum assistance and minimum blood stasis. The blood samples were collected in the order described above to minimize TF contamination of the sodium citrate sample. The sodium citrate tube was carefully inverted after sampling to ensure mixing of the 3.2% trisodium citrate and blood in a 1:9 ratio and kept at room temperature until analysis. The sodium citrate sample was used for TEG analysis, as well as plasma-based coagulation assays. The TEG analysis was performed 30 min after sample collection. The remainder of the sodium citrate sample was centrifuged at 2100 *g* for 8 min, after which the plasma was aliquoted and stored at −80 °C. The EDTA sample was used to perform a CBC and for RLB-PCR. The serum sample was left to clot and then centrifuged at 2100 *g* for 8 min and aliquoted into cryovials. The serum was used to perform a biochemistry profile to identify any complications associated with babesiosis or underlying unrelated disease conditions. The remainder of the serum was stored at −80 °C. Serum aliquots were transported on dry ice to the Veterinary Clinical Pathology Laboratory, University of Copenhagen, for measurement of C-reactive protein (CRP) and serum amyloid A (SAA) concentrations, and transit time for the shipment was less than 48 h.

### 2.3 Complete blood count

A CBC was performed on an automated hematology analyzer (ADVIA 2120, Siemens, Munich, Germany), specifically to measure the hematocrit (HCT), platelet concentration (PLT) and mean platelet volume (MPV) of each dog.

### 2.4 Thromboelastography

The TEG analysis was performed using a thromboelastograph (TEG_®_ 5000 Thrombelastograph® Haemostasis System, Haemonetics Corporation, Braintree, Massachusetts, United States), according to a previously validated method, using TF as activator (28). In short, citrated whole blood was activated with a solution of recombinant human TF (Dade® Innovin®, Siemens, Munich, Germany), prediluted 1:2,780 in HEPES buffer containing 2% bovine serum albumin. The final TF dilution was 1:50,000. Citrated whole blood was left to stand at room temperature for 30 min after collection after which 20 μL CaCl_2_ (280 mM) was added to a prewarmed (37 °C) TEG cup. Subsequently, 25 μL prediluted TF was mixed with 400 μL citrated whole blood, and 340 μL of this premix was added to the cup, resulting in a total volume of 360 μL in each cup. The cup was then gently raised to the pin, and measurements were initiated. Thromboelastograms were obtained for 120 min at 37 °C. Variables recorded from the thromboelstograms included reaction time (R), kinetic time (K), alpha angle (*α*), maximum amplitude (MA), global clot strength (G), fibrinolysis at 30 and 60 min after MA (Ly30 and Ly60), and clot lysis index 30 and 60 min after MA (CL30 and CL60). Variables recorded from the corresponding velocity curve tracing included MRTG, TMRTG and TG. To minimize inter-assay variation, all analyses were performed by the same operator (AG).

### 2.5 Plasma coagulation profile

These assays were performed as a batch and included PT, aPTT, plasma coagulation factor activities (i.e., FII, FVII, FX, FXI), fibrinogen and D-dimer concentrations, and activities for antithrombin (AT), plasminogen and α2-antiplasmin (α2-AP). All assays were performed according to the protocols provided by the manufacturers. The activities of the coagulation factors, plasminogen and α2-AP were assessed using an automated coagulometric analyzer (ACL Elite, Instrumentation Laboratory, Bedford, Massachusetts, United States). A pooled canine control sample, derived from 10 healthy dogs, was run with each batch of tests, and the patient coagulation factor activities were normalized against this pooled control, which was assumed to have a 100% activity. Prothrombin time, aPTT and fibrinogen concentration were measured on the ST art® 4 coagulometric analyzer (Diagnostica Stago, Cedex, France) using the Neoplastine® CI Plus reagent kit for PT, the C. K. Prest® reagent kit for aPTT, and the Sta-Fib 2 reagent kit for fibrinogen concentration according to the manufacturer’s instructions. Plasma AT activity was measured utilizing a thrombin-dependent chromogenic substrate assay (Precimat Chromogen, Roche, Basel, Switzerland) on an automated analyzer (Cobas Integra 400 Plus, Roche, Basel, Switzerland). A normal pooled canine control sample was also run with each batch of tests, and the patient AT activity was normalized against the pooled control sample which was assumed to have a 100% activity. Plasma D-dimer concentration was measured using an immunometric flow-through principle (D-dimer single test, NycoCard READER, Alere, Waltham, Massachusetts, United States) (31). All assays were calibrated according to the manufacturers’ recommendations for human purposes. Commercially available human control reagents for the respective assays were analyzed as internal controls together with pooled plasma from 10 healthy dogs.

### 2.6 Acute phase proteins

The CRP concentration was measured using an automated commercial human turbidimetric immunoassay (CRP, Randox, Crumlin, United Kingdom), calibrated with purified canine CRP (Canine CRP, LifeDiagnostics, West Chester, Pennsylvania, United States). The SAA concentration was measured using an automated latex agglutination turbidimetric immunoassay (SAA-1, Eiken Chemical Company, Tokyo, Japan). Both parameters were analyzed using an automated clinical chemistry analyzer (ADVIA 1800, Siemens, Munich, Germany), and both assays have been validated for use in dogs (32–34).

### 2.7 DNA extraction and PCR

DNA was extracted from 200 μL of each EDTA-anticoagulated whole blood sample using a blood and tissue extraction kit (QIAmp blood and tissue extraction kit, Qiagen, Venlo, The Netherlands) according to the manufacturer’s instructions. Molecular diagnosis of *B. rossi* and exclusion of other *Babesia*, *Theileria, Ehrlichia* and *Anaplasma* species was performed using RLB-PCR. The PCR was conducted with a set of primers that amplified a 460–540 base pair fragment of the 18S SSU rRNA spanning the V4 region, a region conserved for *Babesia* and *Theileria.* The *Ehrlichia* PCR amplified the V1 hypervariable region of the 16S SSU rRNA. The membrane used for RLB included probes for *B. vogeli*, *B. rossi*, *B. canis* and *E. canis* (35).

### 2.8 Statistical analysis

The data were assessed for normality using the Shapiro–Wilk test. The majority of variables were found to be not normally distributed and, therefore, a non-parametric test, the Kruskal-Wallis test, was used to determine significance across groups for each variable. If significance was present, the Mann–Whitney *U* test was used as *post hoc* analysis between groups. Gender proportions were compared using the Chi-square test. Statistical analyses were performed using SPSS 30.0 software (IBM, SPSS Inc., Chicago, Illinois, United States). A *p*-value of *<*0.05 was considered statistically significant.

## 3 Results

### 3.1 Study population characteristics

A total of 106 *Babesia*-infected dogs were enrolled. Nine were excluded due to coinfection with *E. canis* or the presence of comorbidities, including skin wounds, abortion, severe flea infestation or marked verminosis. Ultimately, 97 dogs naturally infected with *B. rossi* and 15 healthy control dogs were included in the study. Of the 97 infected dogs, 12 died (12%) and 85 survived (88%). For the non-survivors, the median time until death (range) was 24 (24–48) hours. The complications and abnormalities observed with the non-survivors included severe anemia, hyperlactatemia, icterus with raised liver enzymes (alanine aminotransferase and alkaline phosphatase >2 times above the highest laboratory reference interval value), hypoglycemia, hypokalemia, neurological signs (seizures or coma in the absence of hypoglycemia), acute pulmonary edema, acute kidney injury, hemoconcentration, and secondary immune mediated hemolytic anemia. There were no significant differences in age or weight between the groups. The median patient age (range) was 20 (3–144) months, with the median age of the survivors 20 (3–144) months and the non-survivors 20.5 (3–79) months. The median age (range) of the control dogs was 48 (3–84) months. The median patient weight (range) was 18.8 (5–65) kg, with the median weight of the survivors 19.4 (5–65) kg and the non-survivors 18.5 (5–40.4) kg. The median weight (range) of the control dogs was 28 (8–65) kg. The ratio of male: female for each group was as follows: controls (5:10), *Babesia*-infected (63:34), survivors (56:29) and non-survivors (7:5). There was a statistically significant difference in sex distribution between the *Babesia*-infected dogs and controls (*p* = 0.02).

### 3.2 Specific hematology variables and acute phase proteins for *Babesia*-infected dogs, survivors and non-survivors, compared to healthy controls at presentation

Table 1 contains a summary of all the descriptive data. Compared to the control dogs, the *Babesia*-infected dogs had a significantly lower HCT (*p* < 0.001) and PLT (*p* < 0.001), as well as a significantly larger MPV (*p* < 0.001). Among survivors and non-survivors, HCT (*p* < 0.001 for both) and PLT (*p* < 0.001 for both) were significantly lower, while MPV was significantly larger (*p* < 0.001 and *p* = 0.002, for survivors and non-survivors respectively), compared to the control dogs. Additionally, HCT was significantly lower in non-survivors compared to survivors (*p* = 0.014). C-reactive protein and SAA concentrations were significantly higher in *Babesia*-infected dogs, including survivors and non-survivors (*p* < 0.001 for all), compared to control dogs. However, no significant differences were observed between survivors and non-survivors for both CRP and SAA concentrations.

**Table 1:** Descriptive statistics for hematology, acute phase proteins, TEG and coagulation profile variables in healthy control dogs and in dogs with babesiosis (survivors and non-survivors) at presentation.

Variable	Unit	Reference interval	Controls	*Babesia*-infected	Survivors	Non-survivors
			Median (IQR)	Median (IQR)	Median (IQR)	Median (IQR)
HCT	L/L	0.37–0.55	0.54 (0.45–0.57)	0.19 (0.13–0.32) ^a^	0.20 (0.14–0.33) ^a^	0.11 (0.09–0.22) ^a,b^
PLT	×10^9^/L	200-500	298 (234–352)	34 (18–58) ^a^	34 (17–59) ^a^	32 (18–52) ^a^
MPV	fL	9.1–12.7	10.8 (8.9–11.8)	15.5 (14.0–16.9) ^a^	15.6 (14.0–17.0) ^a^	15.1 (13.2–16.2) ^a^
CRP	mg/L	0–35	2.6 (2.1–3.8)	111.3 (87.4–154.4) ^a^	111.3 (82.6–154.4) ^a^	116.1 (94.3–178.1) ^a^
SAA	mg/L		14.1 (11.1–15.3)	933.4 (445.5–1610.0) ^a^	813.7 (434.1–1564.4) ^a^	1350.6 (647.8–1896.8) ^a^
R-time	min	2–11	4.1 (3.8–5.1)	7.0 (5.9–8.6) ^a^	7.0 (5.9–8.1) ^a^	7.4 (5.6–9.9) ^a^
K-time	min	1–5	2.1 (1.4–2.7)	2.6 (1.8–4.2) ^a^	2.6 (1.8–4.2) ^a^	2.9 (1.8–4.6)
Angle (α)	degrees	34–77	61.4 (55.2–66.3)	54.8 (40.2–63.0) ^a^	55.0 (40.2–62.3) ^a^	53.3 (42.4–64.7)
MA	mm	42–71	54.1 (51.8–69.6)	51.0 (42.2–61.4) ^a^	51.6 (42.2–61.6) ^a^	50.5 (39.3–60.8)
G-value	dyn/cm^2^	2.4–11.3	5.9 (5.4–11.4)	5.2 (3.7–8.0) ^a^	5.3 (3.7–8.0) ^a^	5.1 (3.3–7.8)
LY30	%	0–6	0 (0–1.7)	0 (0–0.5)	0 (0–0.5)	0 (0–0.5)
LY60	%	0–11	1.9 (0.5–5.7)	0.5 (0–3.0) ^a^	0.6 (0–3.0) ^a^	0.1 (0–2.7)
CL30	%		100 (95.1–100)	100 (97.8–100)	100 (97.8–100)	100 (98.3–100)
CL60	%		92.7 (86.0–95.9)	98.5 (93.2–100) ^a^	98.4 (93.2–100) ^a^	99.3 (90.2–100) ^a^
MRTG	mm/min		10.2 (7.7–14.6)	8.9 (5.5–12.1)	9.1 (5.5–12.1)	7.6 (5.2–13.8)
TMRTG	min		5.3 (4.9–6.6)	8.9 (7.3–11.2) ^a^	8.9 (7.3–11.0) ^a^	9.6 (7.3–12.6) ^a^
TG	mm/min		654.8 (629.0–840.1)	628.0 (551.0–742.2) ^a^	628.0 (551.0–745.3)	624.4 (541.5–740.5)
PT	sec	6.0–9.5	7.0 (6.4–7.4)	7.1 (6.8–7.6)	7.0 (6.7–7.5)	7.6 (7.2–10.0) ^a,b^
aPTT	sec	9.0–12.5	11.7 (11.1–12.4)	13.1 (12.3–14.9) ^a^	13.0 (12.3–14.6) ^a^	13.8 (12.8–16.7) ^a^
Fibrinogen	g/L	2–4	2.1 (1.4–2.6)	4.3 (3.4–5.3) ^a^	4.3 (3.4–5.3) ^a^	4.4 (3.4–5.5) ^a^
AT activity	%	80–150	85.2 (82.8–91.9)	77.2 (67.6–87.3) ^a^	78.1 (71.2–88.0) ^a^	61.6 (56.8–79.3) ^a,b^
D-dimer	mg/L	0-0.5	0.1 (0.1–0.2)	0.2 (0.1–0.5) ^a^	0.2 (0.1–0.4) ^a^	0.4 (0.2–0.5) ^a^
FII	%	80–150	105.8 (84.7–111.1)	78.9 (63.8–95.1) ^a^	81.6 (66.0–96.0) ^a^	68.4 (44.2–91.0) ^a^
FVII	%	80–150	88.9 (84.1–146.0)	74.1 (55.3–104.8) ^a^	77.8 (59.6–111.9) ^a^	49.0 (39.3–73.7) ^a,b^
FX	%	80–120	97.6 (87.3–116.5)	73.6 (50.5–94.6) ^a^	78.3 (58.7–98.4) ^a^	41.5 (28.5–75.2) ^a,b^
FXI	%	80–150	63.2 (53.8–83.6)	41.4 (26.7–62.0) ^a^	44.0 (27.7–61.3) ^a^	36.6 (23.6–75.9) ^a^
Plasminogen	%		134.4 (118.8–172.7)	109.8 (68.7–191.4)	107.1 (68.7–183.4)	140.3 (63.1–239.7)
α2-AP	%		101.0 (93.1–104.8)	111.4 (101.0–127.6) ^a^	113.3 (101.9–129.1) ^a^	101.5 (85.9–116.0)

^a^Significant difference (*p* < 0.05) compared to healthy control dogs. ^b^Significant difference (*p* < 0.05) compared to survivors.

### 3.3 TEG and velocity curve variables for *Babesia*-infected dogs, survivors and non-survivors, compared to healthy controls at presentation

In *Babesia*-infected dogs, the R-time (*p* < 0.001) and K-time (*p* = 0.021) were significantly longer, while the *α*-angle (*p* = 0.015), MA (*p* = 0.027) and G (*p* = 0.027) were significantly smaller compared to control dogs. Although no significant differences were observed for LY30 and CL30, the LY60 was significantly lower (*p* = 0.037) and the CL60 significantly higher (*p* < 0.001) in the *Babesia*-infected dogs. Based on velocity curve analysis, mean MRTG was not significantly different for the *Babesia*-infected dogs, compared to the control dogs. However, TMRTG was significantly longer (*p* < 0.001), and TG was significantly lower (*p* = 0.048).

Among survivors, the R-time (*p* < 0.001) and K-time (*p* = 0.023) were significantly longer, *α*-angle (*p* = 0.015), MA (*p* = 0.032) and G (*p* = 0.032) were significantly smaller, and LY60 was significantly lower (*p* = 0.049) and CL60 significantly higher (*p* < 0.001) compared to control dogs. In non-survivors, only R-time was significantly longer (*p* < 0.001) and CL60 significantly higher (*p* = 0.024) compared to the control dogs. Based on the velocity curve analysis, TMRTG was significantly longer in both the survivors and non-survivors (*p* < 0.001 for both), while there were no significant differences observed between survivors and non-survivors for any of the TEG and velocity curve variables.

### 3.4 Plasma-based hemostatic variables for *Babesia*-infected dogs, survivors and non-survivors, compared to healthy controls at presentation

**Figure 1:**
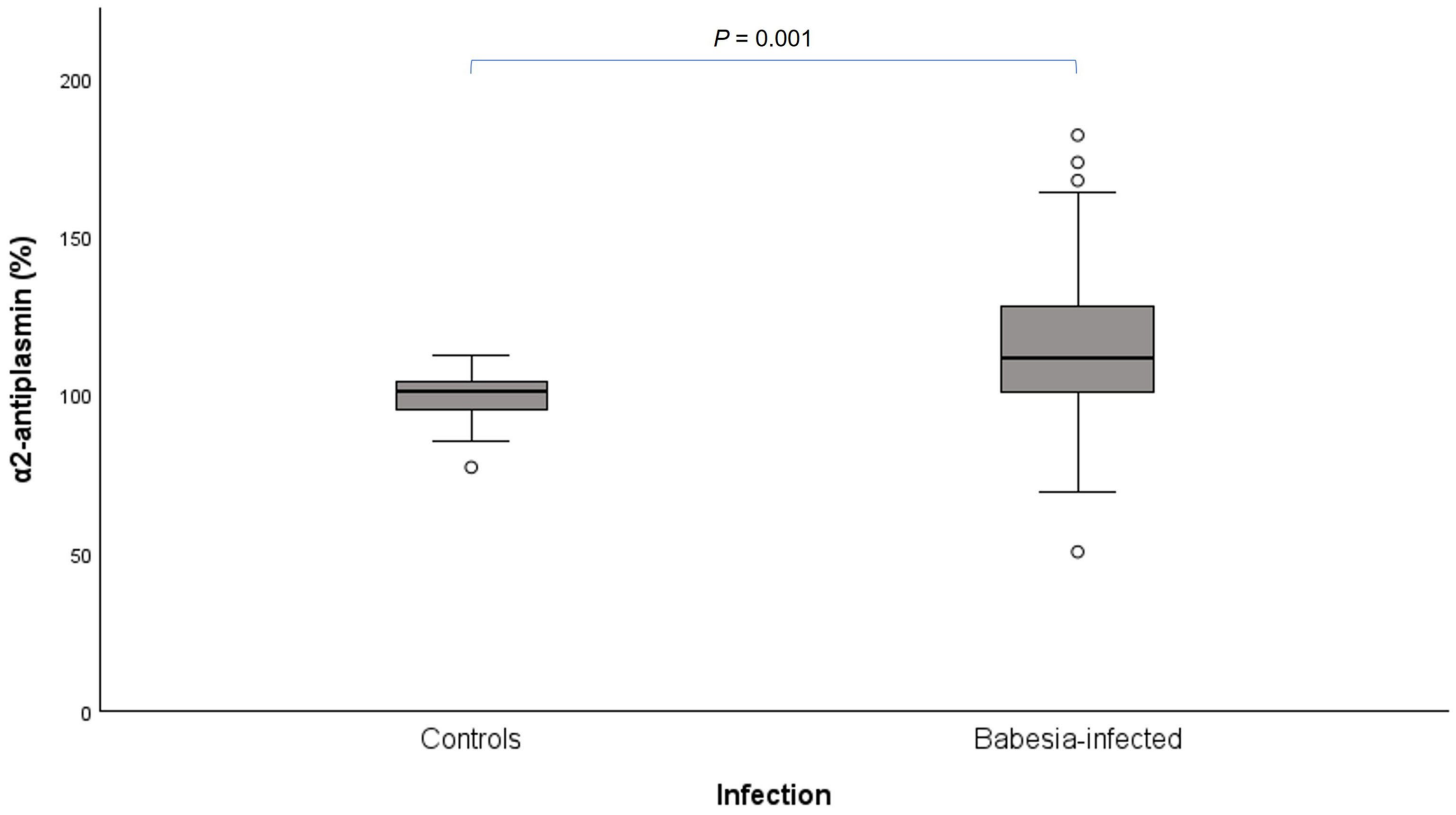
Box plot of α2-antiplasmin activity in the *Babesia*-infected group compared to the healthy control group. Key for interpretation of box plots: The box represents the IQR (i.e., the middle 50% of the observations) with the line inside the box as the median. The whiskers represent the main body of the data, indicating the range of the data. Outliers, values that are 1.5 times removed from the IQR, are plotted as open circles. Extreme outlier values that are 3 times removed from the IQR are represented by asterisks.

In *Babesia*-infected dogs, the activities of FII (*p* < 0.001), FVII (*p* = 0.006), FX (*p* < 0.001) and FXI (*p* = 0.002) were significantly lower, compared to the control dogs. However, only aPTT was significantly longer (*p* < 0.001). Fibrinogen (*p* < 0.001) and D-dimer (*p* = 0.033) concentrations were significantly higher and AT activity significantly lower (*p* = 0.006) in *Babesia*-infected dogs compared to control dogs. There were no significant differences between *Babesia*-infected and control dogs for plasminogen activity, whereas α2-AP activity was significantly higher (*p* = 0.001) in *Babesia*-infected dogs (Figure 1).

**Figure 2:**
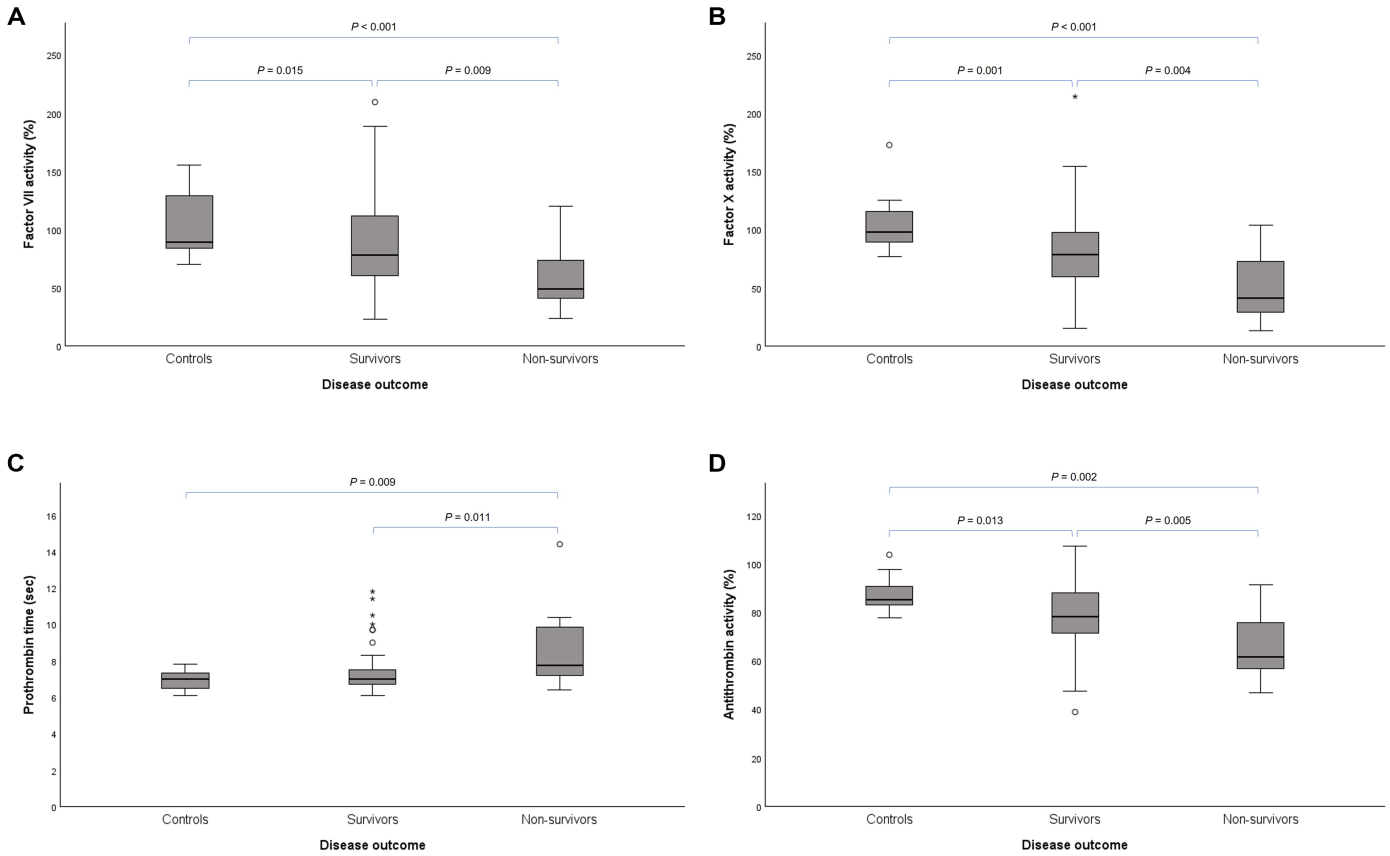
Box plots of **(A)** factor VII activity, **(B)** factor X activity, **(C)** prothrombin time, and **(D)** antithrombin activity illustrating significantly lower procoagulant and anticoagulant activities and prolonged clotting time in the non-survivors compared to the survivors and healthy control group.

Among survivors, the activities for FII (*p* < 0.001), FVII (*p* = 0.015; Figure 2A), FX (*p* = 0.001; Figure 2B), and FXI (*p* = 0.002) were significantly lower compared to control dogs. Only aPPT was significantly longer (*p* < 0.001). In non-survivors, these same factor activities were significantly reduced, namely FII (*p* < 0.001), FVII (*p* < 0.001; Figure 2A), FX (*p* < 0.001; Figure 2B), and FXI (*p* = 0.021), with both PT (*p* = 0.009; Figure 2C) and aPTT (*p* < 0.001) significantly longer compared to control dogs. Among the survivors and non-survivors, fibrinogen concentration (*p* < 0.001 and *p* = 0.002, respectively) and D-dimer concentration (*p* = 0.043 and *p* = 0.024, respectively) were significantly higher, while AT activity was significantly lower (*p* = 0.013 and *p* = 0.002, respectively; Figure 2D), compared to control dogs. The α2-AP activity was significantly higher in survivors (*p* < 0.001) compared to the control dogs. Compared to survivors, non-survivors had significantly lower activities for FVII (*p* = 0.009; Figure 2A) and FX (*p* = 0.004; Figure 2B), as well as a significantly longer PT (*p* = 0.011; Figure 2C). Additionally, AT activity was significantly lower in non-survivors compared to survivors (*p* = 0.005; Figure 2D).

### 3.5 Post mortem and histopathology findings

**Figure 3:**
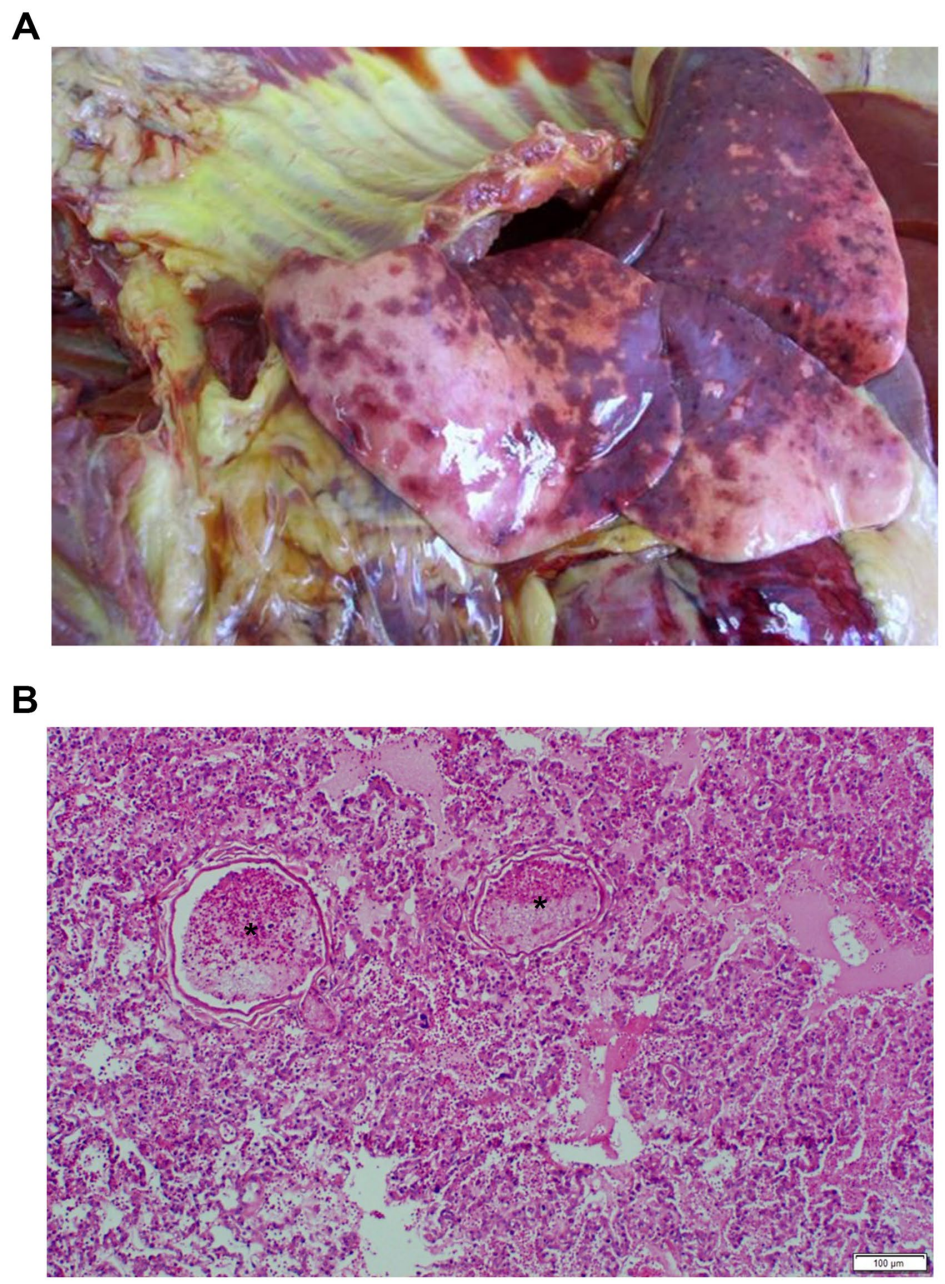
**(A)** Macroscopic lesions at necropsy indicating pulmonary congestion, edema, and atelectasis with multifocal areas of hemorrhage, as well as **(B)** microscopic evidence of the presence of fibrin microthrombi (asterisks) within small vessels of the lung (H&E processing) of a *Babesia*-infected dog that did not survive.

Of the 12 dogs that died, eight had macroscopic and/or microscopic evidence of microthrombi. Organs that were typically affected included the heart, lungs, spleen, liver, kidneys, and brain, as well as the mesentery. Macroscopic findings included moderate to severe myocardial hemorrhages and infarcts; pulmonary congestion, edema, and atelectasis with multifocal areas of hemorrhage (Figure 3A); splenomegaly secondary to red and white pulp hyperplasia, with multiple infarcts; and severe, multifocal hemorrhage and necrosis of the cerebral cortex. Microscopic findings included the presence of severe leukostasis with fibrin strand formation, as well as the presence of fibrin microthrombi within small and larger blood vessel (Figure 3B).

## 4 Discussion

This study demonstrated that dogs with virulent babesiosis exhibit marked hemostatic dysfunction, which was more severe in non-survivors. Abnormalities included thrombocytopenia, platelet activation, consumption of coagulation factors, and fibrinolytic shutdown, alongside a pronounced systemic inflammatory response. Collectively, these findings support the presence of DIC with microthrombus formation, likely driven by thromboinflammatory mechanisms, with greater severity observed in dogs that did not survive. Furthermore, the minimal fibrinolytic activity observed, may have contributed to the persistence of a prothrombotic state and the increased mortality observed in affected dogs.

The immune and coagulation systems are closely interconnected, working together to support host defense. However, when systemic inflammation is prolonged or the inflammatory response becomes excessive, this regulated process can shift into thromboinflammation, driven by dysregulated immunothrombosis. This shift triggers platelet activation and aggregation, as well as leukocyte recruitment, leading to endothelial injury and progression to pathological states such as DIC, multiple organ failure, and death (3, 8). In this setting, thrombosis and inflammation not only worsen outcomes independently but also amplify each other’s effects.

In this study, infected dogs showed markedly increased CRP and SAA concentrations, confirming systemic inflammation. Dogs naturally infected with *B. rossi* have been reported to mount a marked proinflammatory response, reflected by increased cytokines and acute phase proteins in the blood. In particular, interleukin (IL)-6, IL-10, and monocyte chemotactic protein-1 have been shown to be significantly higher in dogs that succumbed to the disease (18, 19). Pathogens that induce systemic inflammation are known to activate platelets, a phenomenon previously reported in *B. rossi* infection (20, 21, 36). Interestingly, despite the severe thrombocytopenia observed in babesiosis, affected dogs typically do not display a clinically evident bleeding phenotype at presentation. This is likely due to heightened platelet activation providing a procoagulant surface (20, 37). Upon activation, platelets undergo morphological changes, release granule contents, and upregulate the expression of key surface receptors, including glycoprotein (GP) Ib-IX-V complex, which mediates vWF-dependent primary platelet adhesion; P-selectin, which facilitates interactions with leukocytes and other platelets; and GP IIb/IIIa, the fibrinogen receptor essential for platelet aggregation and secondary thrombus formation (4, 38, 39). Increased MPV, an indirect marker of platelet activation, reflects the release of young, highly reactive platelets from the bone marrow in response to increased platelet consumption or inflammation. Larger platelets are more prone to aggregation and secretion, promoting a prothrombotic state (40). Similar to previous findings (20), infected dogs in this study had significantly higher MPV values compared to healthy controls, with no difference detected between survivors and non-survivors.

Beyond their classical role in hemostasis, platelets are central to immunothrombosis through various interconnected mechanisms. Activated platelets release alpha granule contents, which recruit monocytes and neutrophils, forming platelet-leukocyte aggregates. These aggregates enhance TF expression and promote NET formation, creating a self-perpetuating prothrombotic cycle (1, 8, 9). For some pathogens, immune cell recognition may amplify TF activation >100 fold (7). In dogs with *B. rossi* infection, increased platelet-leukocyte aggregates, specifically platelet-monocyte aggregates, have been described and may be a good indicator of thromboinflammation in inflammatory disease (21). Platelet-leukocyte interactions enable bidirectional immune crosstalk and transactivation, contributing to vascular inflammation (9). The interaction between activated platelets and leukocytes enhances the expression of TF, especially in monocytes. Monocytes are key producers of inducible intravascular TF on their cell surface or in the form of microvesicles. Tissue factor upregulation plays a pivotal role in immunothrombosis and, if uncontrolled, drives thromboinflammation (1, 8). Mature neutrophils have the greatest capacity for NET production, and platelet–neutrophil interactions facilitate NETosis (1, 8, 9, 41).

In *Babesia*-infected dogs, clot initiation was prolonged and clot propagation slowed compared to healthy control dogs, based on TEG variables R, K, *α*-angle, and TMRTG. No significant differences were observed between survivors and non-survivors. For all groups, the median values for these variables fell within the laboratory reference intervals. Although TEG is influenced by both factor concentration and activity, it is generally insensitive to mild deficiencies, which may explain the absence of significant differences between survivors and non-survivors in this study (42, 43). Because conventional TEG variables are non-parametric, MRTG may provide a more accurate measure of clot propagation (44, 45). However, MRTG in the *Babesia*-infected dogs was not significantly different from that of the healthy controls, a finding similar to that reported in dogs with parvoviral enteritis (30). The prolonged R correlated with the increased PT and aPTT times, as well as the markedly reduced plasma coagulation factor activities in the *Babesia*-infected dogs; changes that were more pronounced in those that died. In the non-survivors, FVII and FX activities were significantly lower compared to the survivors, explaining why PT (reflecting the extrinsic and common pathway) was significantly longer in this group compared to the survivors and control dogs. These reductions in clotting factor activity likely reflect more severe inflammation-induced coagulation activation and factor consumption caused by the infection (13, 24, 25). In addition, thrombin (FIIa) and other factors such as FVIIa and FXa can activate multiple cell types, triggering intracellular inflammatory pathways (46). This may create another self-perpetuating cycle of inflammation-driven factor consumption in the non-survivors. Consumptive coagulopathy, marked by procoagulant activation, depletion of clotting factors, and loss of natural inhibitors, has previously been reported in *B. rossi* infection and linked to increased mortality (13).

Antithrombin activity was markedly decreased in the *Babesia*-infected dogs in this study, with the lowest activities observed in non-survivors. As a key endogenous anticoagulant, AT inhibits clotting factors across the extrinsic, intrinsic, and common pathways (47). Beyond its anticoagulant function, AT also modulates inflammation; directly by influencing endothelial anti-inflammatory activity, and indirectly by inhibiting proinflammatory coagulation enzymes such as thrombin and FX (48). In human COVID-19 patients, where it has been established that thrombotic complications play an important role in disease pathogenesis and outcome, reduced AT activity has similarly been associated with persistent hypercoagulability and a proinflammatory state in fatal cases, highlighting its central role in the pathogenesis of thromboinflammatory disease (49).

**Figure 4:**
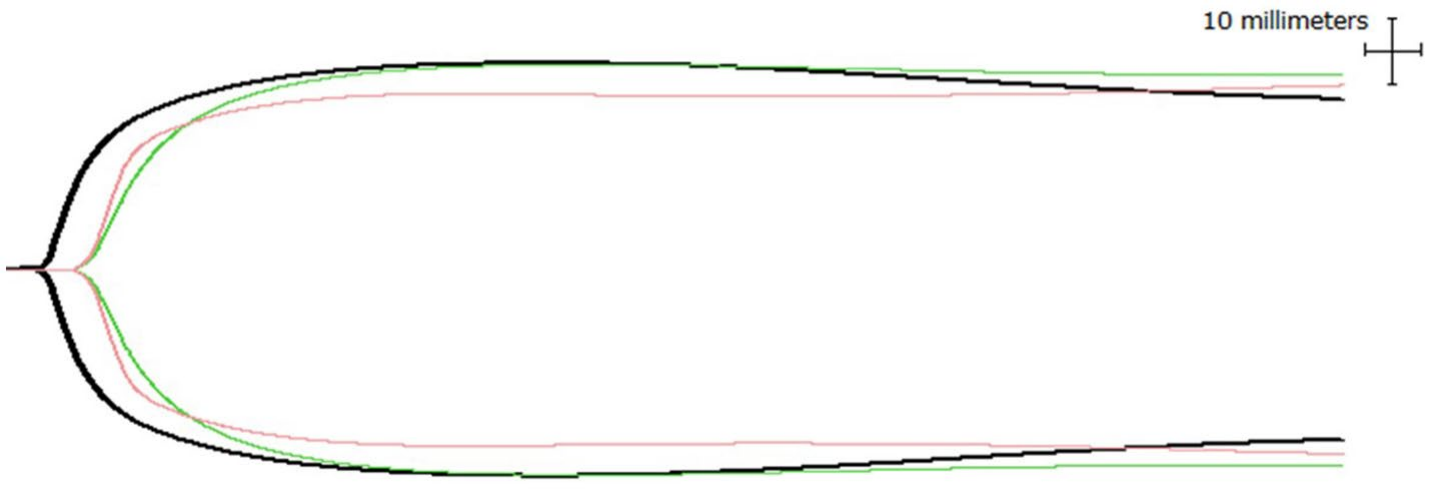
Selected thromboelastogram tracings of a dog from each group representative of the median values for each variable; healthy control (black), survivor (pink), and non-survivor (green).

Compared with the healthy control dogs, the *Babesia*-infected dogs had reduced clot strength, as indicated by lower MA, G, and TG values. Thrombocytopenia of the severity typically observed in canine babesiosis (37) would normally produce markedly hypocoagulable TEG tracings, characterized by longer K, smaller *α*-angle, and lower MA (50–52). Interestingly, in this cohort, the median MA values in *Babesia*-infected dogs remained within the normal laboratory reference interval. Interpretation of MA in these dogs is complicated by anemia, as MA is inversely correlated with HCT (50, 51, 53). Nonetheless, a prior study in uncomplicated babesiosis found no correlation between MA/G and HCT or fibrinogen, but did observe a relationship between MA/G and PLT (23). Results from previous studies suggest that severe thrombocytopenia in babesiosis is offset by the combined effects of activated platelets, expressing an abundance of GP IIb/IIIa on their surfaces, pronounced hyperfibrinogenemia, and the resulting fibrin contribution to clot strength, leading to a normocoagulable TEG profile (Figure 4) and absence of clinical bleeding (22, 54). The hyperfibrinogenemia observed is likely as a result of the intense acute phase inflammatory response characteristic of canine babesiosis, supported by the extremely high serum CRP and SAA concentrations in this study (55, 56).

Fibrinolysis is initiated during coagulation when plasminogen is converted to plasmin by tissue-type plasminogen activator (tPA) and urokinase plasminogen activator. Plasmin degrades fibrinogen and cross-linked fibrin, generating D-dimer (57). The process is tightly regulated by the balance between tPA and plasminogen activator inhibitor-1 (PAI-1), both released from endothelial cells. Thrombin generation or endothelial injury stimulates tPA release, promoting fibrinolysis; however, this is rapidly counterbalanced by increased endothelial production of PAI-1. Increased PAI-1 levels, characteristic of thromboinflammation, tip the balance toward hypofibrinolysis, a phenomenon termed “fibrinolytic shutdown” (2). Neutrophil extracellular traps further impair fibrinolysis, as DNA-bound elastase reduces plasminogen activation and forms inhibitory complexes with plasmin and fibrin (58).

In this study, the *Babesia*-infected dogs showed minimal fibrinolysis 60 min after maximum clot strength was reached, evidenced by significantly lower LY60 and higher CL60 compared to healthy controls, with no difference between survivors and non-survivors. Although D-dimer concentrations were significantly higher in the *Babesia*-infected dogs, survivors and non-survivors, compared to the healthy control dogs, the values in the *Babesia*-infected dogs remained within the laboratory reference interval. There was also no difference between survivors and non-survivors, which is in contrast with previous findings where non-survivors had increased D-dimer concentrations (13). Since D-dimer reflects degradation of cross-linked fibrin, increased concentrations would indicate ongoing fibrin formation (57). While correlations between D-dimer and TEG fibrinolysis variables have been documented in humans, such associations are less clear in veterinary medicine (27, 59). Routine TEG assays are relatively insensitive for detecting hypofibrinolysis or predicting thrombotic risk and should be modified with exogenous tPA to better assess fibrinolytic impairment. In fact, studies in dogs with prothrombotic conditions such as necrotizing pancreatitis, protein-losing nephropathy, or mammary carcinoma have demonstrated greater resistance to tPA-induced fibrinolysis than healthy controls (60). Increased thrombin-activatable fibrinolysis inhibitor activity has also been documented in septic dogs (61). In humans, traditional fibrinolysis biomarkers such as α2-AP are considered more sensitive, as TEG only detects fibrinolysis when tPA levels are markedly increased (62). α2-Antiplasmin is the principal ultrafast, covalent inhibitor of plasmin and a member of the serine protease inhibitor (serpin) family. Synthesized primarily by hepatocytes, it serves as a key regulator of fibrinolysis. Activated FXIII covalently cross-links α2-AP to fibrin within the thrombus, a process shown to markedly increase fibrin resistance to plasmin-mediated degradation. In humans, elevated α2-AP activity is significantly associated with an increased risk of cardiovascular and cerebrovascular thrombotic events (63, 64). Although plasminogen activity did not differ significantly between groups in this study, α2-AP activity was significantly higher in *Babesia*-infected dogs, supporting reduced fibrinolysis in this cohort. Circulating α2-AP is normally present at approximately half the plasma concentration of its target zymogen, plasminogen (64). Therefore, increased α2-AP activity in the face of “normal” plasminogen activity in dogs with babesiosis warrants further investigation as a sequela of thromboinflammation. The presence of fibrin microthrombi in multiple organs of non-survivors further corroborates a hypercoagulable, prothrombotic state in canine babesiosis.

There were several limitations to this study. The *Babesia*-infected dogs presented at different stages of the disease process, likely contributing to variability in results based on the duration of illness prior to presentation. Although TEG provides a holistic view of coagulation, it does not include an important component of hemostasis, namely the endothelium, which represents a methodological limitation and may have influenced the results in the various groups. Despite this, a consistent pattern of hemostatic dysfunction was observed in *Babesia*-infected dogs. In addition, cases were managed by different clinicians resulting in variability in treatment approaches, which may have influenced outcomes. Finally, this study was also limited by the number of assays available for fibrinolysis analysis.

In conclusion, dogs naturally infected with *B. rossi* show a marked interplay between systemic inflammation and coagulation, characterized by thrombocytopenia, platelet activation, coagulation factor consumption, altered clot dynamics, and impaired fibrinolysis, culminating in a prothrombotic state associated with poor outcomes. These findings indicate that dysregulated immunothrombosis in canine babesiosis progresses to thromboinflammation, driving endothelial injury and microthrombus formation, thereby predisposing to organ failure and death. While this study was not designed to develop or validate diagnostic or prognostic tools for thromboinflammation in dogs, the findings provide insight into relevant pathophysiological processes and may inform future research efforts that could ultimately contribute to the development of clinically applicable tools, with potential benefits for clinical decision-making, therapeutic interventions and patient outcomes.

## Data Availability

The raw data supporting the conclusions of this article will be made available by the authors, without undue reservation.
